# Viral infections in hospitalized children in Germany during the COVID-19 pandemic: Association with non-pharmaceutical interventions

**DOI:** 10.3389/fped.2022.935483

**Published:** 2022-08-11

**Authors:** Nicolas Terliesner, Nadine Unterwalder, Anke Edelmann, Victor Corman, Andreas Knaust, Leonard Rosenfeld, Alexander Gratopp, Hannelore Ringe, Luise Martin, Horst von Bernuth, Marcus A. Mall, Tilmann Kallinich

**Affiliations:** ^1^Department of Pediatric Respiratory Medicine, Immunology and Critical Care Medicine, Charité-Universitätsmedizin Berlin, Corporate Member of Freie Universität Berlin, Berlin Institute of Health, Humboldt-Universität zu Berlin, Berlin, Germany; ^2^Labor Berlin GmbH, Berlin, Germany; ^3^Charité-Universitätsmedizin Berlin, Corporate Member of Freie Universität Berlin, Berlin Institute of Health, Institute of Virology, Humboldt-Universität zu Berlin, Berlin, Germany; ^4^Berlin Institute of Health at Charité–Universitätsmedizin Berlin, Berlin, Germany; ^5^Charité-Universitätsmedizin Berlin, Corporate Member of Freie Universität Berlin, Berlin Institute of Health, Berlin-Brandenburg Center for Regenerative Therapies, Humboldt-Universität zu Berlin, Berlin, Germany; ^6^Deutsches Rheumaforschungszentrum, An Institute of the Leibniz Association, Berlin, Germany

**Keywords:** COVID-19, pandemic, child, Bocavirus, RSV, non-pharmaceutical interventions (NPI), respiratory virus infection, gastrointestinal infection

## Abstract

**Background:**

Non-pharmaceutical interventions (NPI) during the COVID-19 pandemic aimed at prevention of SARS-CoV-2 transmission also influenced transmission of viruses other than SARS-CoV-2. The aim of this study was to describe and compare the burden of common viral respiratory and gastrointestinal infections in children admitted to Berlin University Children's Hospital (BCH) before and during the COVID-19 pandemic at different levels of public NPI measures.

**Methods:**

In this retrospective study, we analyzed the frequency of detection of common human respiratory and gastrointestinal viruses from January 2016 through January 2022 in all patients admitted to BCH. We compared virus detection before and during the COVID-19 pandemic at different levels of public NPI measures.

**Results:**

The frequency of detection of seasonal enveloped and non-enveloped viruses [Boca-, Corona-, Influenza-, Metapneumo-, Parainfluenza-, Rota-, and Respiratory Syncytial Viruses (RSV)] was diminished during the COVID-19 pandemic, whereas detection rates of non-seasonal viruses (Rhino-/Entero-, and Adenoviruses) were stable during the pandemic. After withdrawal of major NPI measures, we observed an out of season surge of the detection rates of Boca-, Corona-, Parainfluenzaviruses, and RSV. In contrast, no increased detection frequency was observed for Influenza-, Metapneumo-, and Rotaviruses as of January 2022.

**Conclusion:**

Corona-, Boca-, Parainfluenzaviruses, and RSV returned as frequently detected pathogens after withdrawal of major NPI measures. The out of season rise might be attributed to an “immune-debt” due to missing contact to viral antigens resulting in waning of population immunity during the COVID-19 pandemic.

## Introduction

**Figure 1:**
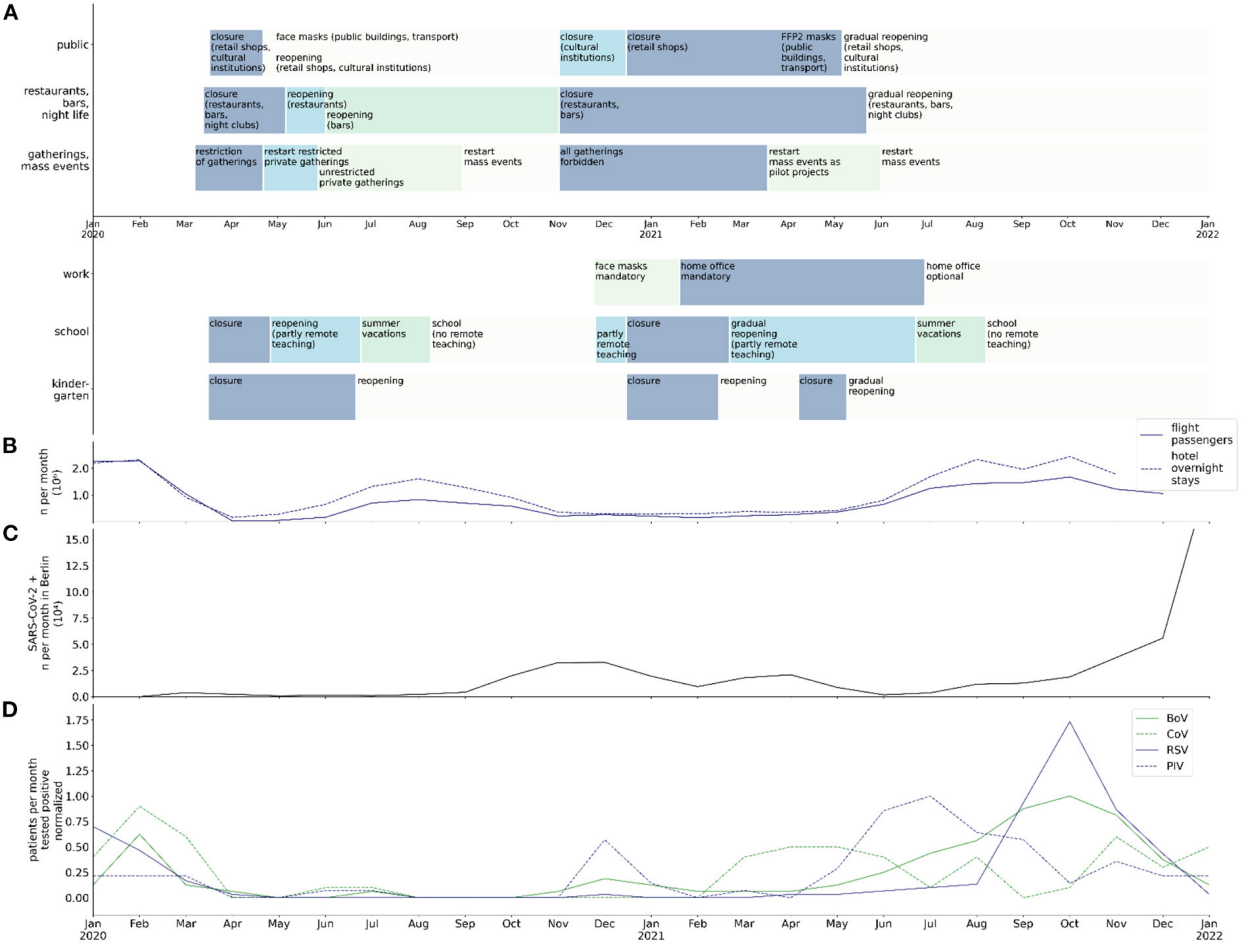
Chronology of NPI, traveling, SARS-CoV-2 detection rate in Berlin, and normalized virus detection rate at BCH between January 2020 and January 2021; **(A)** chronology of public regulations in public buildings/spaces, restaurants and night life, private and public gatherings, workplace, school, and kindergartens in Berlin; **(B)** numbers (10^6^) of flight passengers and overnight stays in hotels per month in Berlin; **(C)** rate of SARS-CoV-2 infections (10^4^ per month) in Berlin; **(D)** max-normalized rate of infections (per month) with BoV, CoV, RSV, and PIV in hospitalized patients at BCH.

Since 2020 countries around the world focused on the mitigation of effects of the COVID-19 pandemic to their population (1). The paramount goal was to reduce morbidity and mortality by infection with SARS-CoV-2. During 2020, when there were no vaccines against SARS-CoV-2 available, countries imposed non-pharmaceutical interventions (NPI) that aimed at the prevention of virus transmission. In Germany, mass gatherings were prohibited, working remotely became mandatory where feasible, schools and kindergartens were closed as well as retail shops, restaurants, bars, and cultural institutions (Figure 1A). Restrictions on border crossing and associated quarantine measures led to a decrease in traveling activity (Figure 1B). Use of face masks became mandatory in public areas, buildings and public transport. Quarantine became obligatory in cases of infection or an increased risk of infection with SARS-CoV-2. Parts of these measures were temporary and corresponded to dynamics of the local and countrywide incidence of SARS-CoV-2 infection (Figure 1C). Other measures were still in place in January 2022, such as the use of face masks in public buildings and public transport.

On December 21, 2020, the European Medicines Agency (EMA) recommended the first COVID-19 vaccine for authorization in Europe and within 1 week vaccination was commenced in Germany (2). From then on until mid-2021 NPI measures were gradually lifted, corresponding to vaccination rates and to dynamics of incidence of infection with SARS-CoV-2 and hospital capacities. COVID-19 immunization was initially not approved for children. Only a small part of the pediatric population, adolescents from an age of 12 years, was immunized against SARS-CoV-2 in Germany, starting on August 16, 2021. The fear of transmission of SARS-CoV-2 among children attending school or kindergarten led to governmental directives enforcing closures of schools and kindergartens, in contrast to workplaces (3). In Berlin, kindergartens were finally reopened in May 2021, in-classroom teaching for all students was commenced after the 2021 summer holidays, in August 2021.

While NPI measures during the COVID-19 pandemic focused on the prevention of the transmission of SARS-CoV-2, it was likely that NPI measures also affected the transmission of other, airborne/droplet or fecal-orally transmitted viral pathogens. As major NPI measures were lifted in Germany during spring and summer of 2021, their preventive effect on infections other than SARS-CoV-2 might have been suspended too. This may have led to a recurrence of common viral infections in children and adolescents. Yet it was unclear whether the long period of NPI measures affected the population immunity to common pathogens and the presence of these pathogens in Berlin and Germany.

The aim of this study was therefore to determine the frequency of common airborne/droplet or fecal-orally transmitted viral infections in hospitalized children and adolescents at BCH, the largest children's university hospital in Germany. We compared the rates of detection of viral pathogens in hospitalized children and adolescents before and during the COVID-19 pandemic at different levels of public NPI measures.

## Materials and methods

### Study design

We undertook a retrospective longitudinal study to compare monthly rates of detection of common viral respiratory and gastrointestinal tract infections in children admitted to BCH before the COVID-19 pandemic and during the pandemic at different levels of public NPI measures. During the observation period from January 2016 to January 2022 admitted patients with symptoms of a respiratory tract infection regularly underwent diagnostic testing for respiratory viruses [human Bocavirus (BoV), Coronaviruses 229E, NL63, HKU1, and OC43 (CoV), Influenzaviruses A, B (InV), human Metapneumovirus (MPV), Parainfluenzaviruses 1-4 (PIV), Respiratory Syncytial Viruses A, B (RSV), Rhino-/Enteroviruses (RV/EV)], as well as SARS-CoV-2 (since 2020) using a PCR-based Respiratory Pathogen Panel. Admitted patients with diarrhea regularly underwent testing for Rotavirus (RoV) by an enzyme immuno assay (EIA) or by Reverse Transcription-Polymerase Chain Reaction (RT-PCR), and Adenoviruses (AdV) by PCR. We analyzed all results obtained by Respiratory Pathogen Panel testing, all Rotavirus EIA/RT-PCR and Adenovirus PCR results of all children (age < 18 years), regardless of gender and comorbidities, admitted to BCH during the observation period. As the first period of social and hygiene restrictions (“lockdown”) in Berlin began on March 14, 2020, we defined the beginning of April 2020 as the beginning of the pandemic period in terms of our analyses concerning the frequency of common respiratory and gastrointestinal tract viruses.

### Sampling and laboratory analyses

The decision for diagnostic testing of inpatients was made by the physician in charge based on the patient's respiratory or gastrointestinal symptoms. Outpatients were not tested. In spontaneously breathing patients, an oropharyngeal swab was performed and analyzed. In mechanically ventilated patients we analyzed tracheal secretion or an oropharyngeal swab. Nucleic acids were isolated from 200 μl respiratory sample material using the MagNA Pure 96 instrument (Roche Diagnostics, Germany) according to the manufacturer's instructions. Amplification and detection of nucleic acids from respiratory pathogens were performed using the qualitative test NxTAG® Respiratory Pathogen Panel as well as NxTAG® Respiratory Pathogen Panel + SARS-CoV-2 (NxTAG® RPP + SARS-CoV-2; Luminex Corporation, USA). The NxTAG workflow included multiplex RT-PCR and bead hybridization in a thermocycler (GeneAmp 9700, Applied Biosystems, USA) followed by read on the MAGPIX System (Luminex Corporation, USA). The limit of detection (LoD) titer for each of the NxRPP targets was defined as the lowest concentration at which ≥95% of the samples tested generated positive calls and was reported of 10^2^ to 10^4^ copies/ml. Detection of AdV DNA was performed after manual isolation of nucleic acids from 200 μl stool suspension (QIAamp DNA Mini Kit, Qiagen, Germany) followed by real-time PCR according to Heim et al. (4) on LightCycler® 2.0 (Roche Diagnostics, Germany). The LoD of the AdV target in the in-house assay is 2,000 copies/ml. All positive results, whether weakly or strongly positive, were classified as a virus detection.

### Data collection and analysis

Data were generated and analyzed using the instrument specific software, obtained results were stored in the laboratory information system and analyzed using Python 3.7 and its libraries (open source). We excluded all tests with an equivocal result. To avoid a possible bias by multiple testing during an ongoing infection of the same patient with the same virus, we included only the first positive PCR or EIA result of each virus from every 8 weeks interval of each patient. We excluded all viruses detected <60 times during the observation period from our analyses. This excluded Adenovirus (throat swab PCR) and Norovirus (stool PCR). Flight passenger statistics in Berlin were derived from the official website of Berlin Brandenburg airport. Statistics on overnight hotel stays in Berlin were derived from destatis, the official website of the Federal Statistical Office (Statistisches Bundesamt) of the Federal Republic of Germany.

We used Shapiro-Wilk test to test for normal distribution of monthly virus detection rates. Only the monthly detection rate of RV/EV detection followed normal distribution. Consequently, we used a two-tailed student's *t*-test to test for statistical significance of the observed results concerning RV/EV. To test for statistical significance of the observed results concerning all other viruses, we used a two-tailed Mann-Whitney *U* test. Ethical review or approval were not required for this study.

## Results

### Detection of respiratory viral pathogens

**Figure 2:**
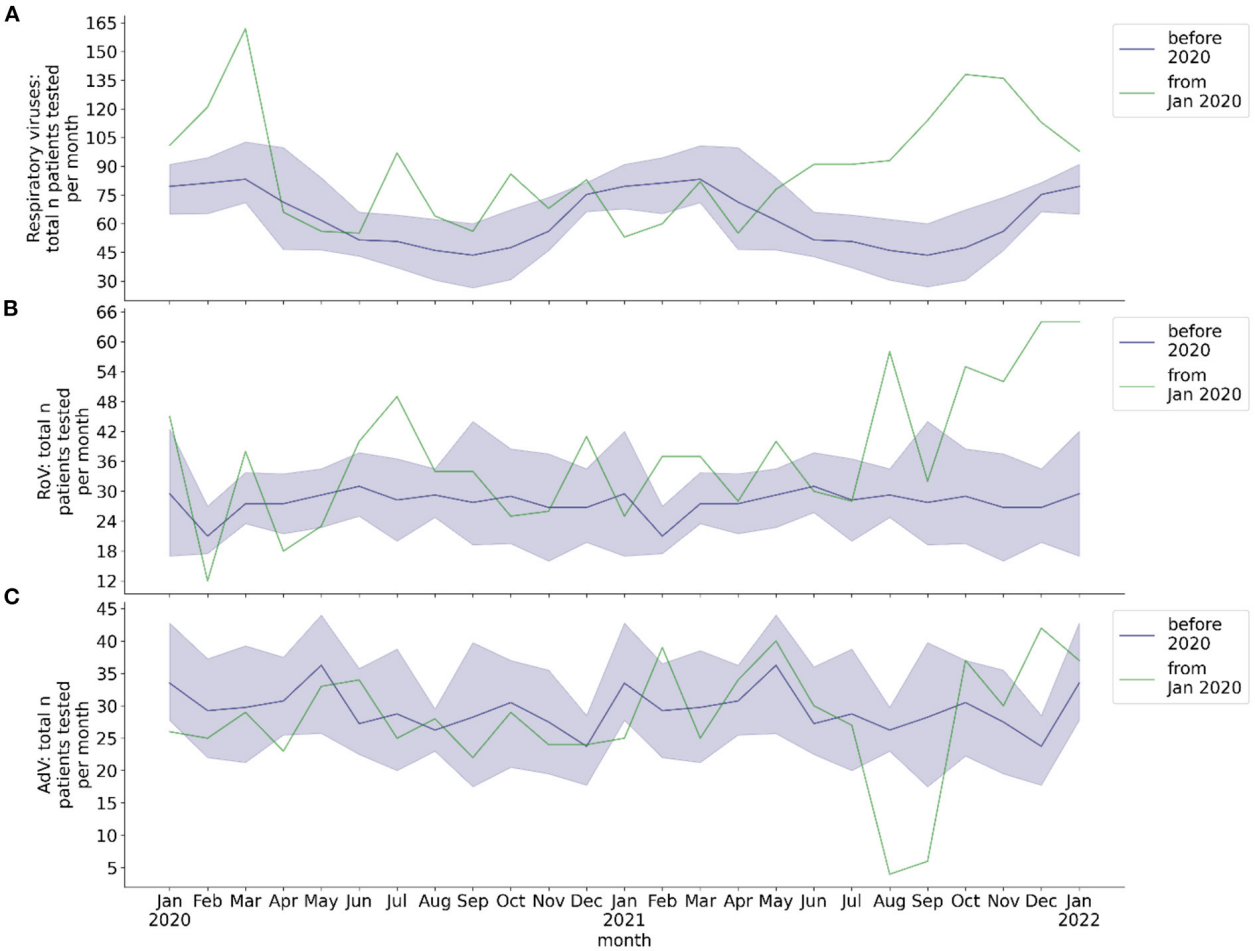
Total monthly number of hospitalized patients at BCH tested for respiratory viruses and for RoV and AdV, respectively. Blue line: mean 2016-2019, blue shade: 95% confidence interval 2016-2019, green line: absolute number per month from 2020, calendar months 2016-2019 are shown as a historical reference and correspond to calendar months, not to year on x-axis. **(A)** Total number of patients per month with molecular biology testing of respiratory samples for respiratory viruses; **(B)** total number of patients per month with EIA or molecular biology testing of stool sample for RoV; **(C)** total number of patients per month with molecular biology testing of stool sample for AdV.

The total number of analyzed respiratory samples was 5,237, total number of patients tested for respiratory viruses was 3,159. The mean total monthly number of molecular biology testing of throat swabs from hospitalized patients was 66.2 before the COVID-19 pandemic, 68.5 from the beginning of the pandemic through May 2021, and 109.3 from June 2021 to January 2022 (Figure 2A).

#### Viral positive respiratory specimens per month before and during COVID-19 pandemic

**Figure 3:**
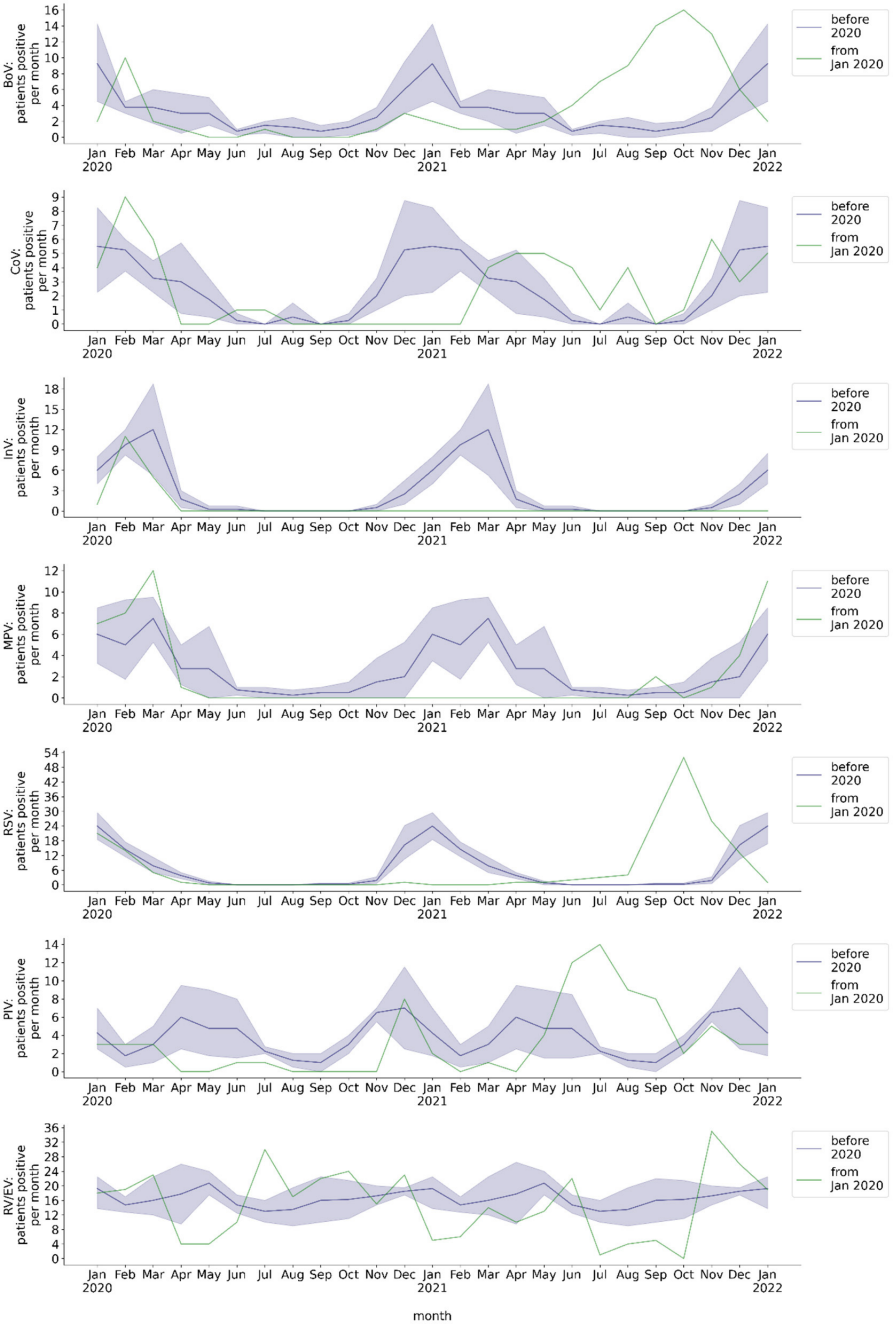
Monthly number of hospitalized patients at BCH positively tested for respiratory viruses. Blue line: mean 2016-2019, blue shade: 95% confidence interval 2016-2019, green line: absolute number per month from 2020, calendar months 2016-2019 are shown as a historical reference and correspond to calendar months, not to year on x-axis. Number of patients per month with respiratory sample tested positive for BoV, CoV, InV, MPV, RSV, PIV, or RV/EV.

The pre-pandemic monthly number of throat swabs of hospitalized patients tested positive by RT-PCR showed a seasonal behavior of BoV, CoV, InV, MPV, and RSV detection (Figure 3). It showed a two peaked seasonality of PIV detection. In contrast, there was no seasonality of RV/EV. From the beginning of the COVID-19 pandemic in Germany through May 2021, several viruses were no longer detected by RT-PCR or the number of monthly positive throat swabs was substantially diminished (Figure 3). This applied to InV (whole year, monthly mean: 2.92 pre-pandemic, 0 during pandemic, *p* = 0.001), MPV (whole year, monthly mean: 2.88 pre-pandemic, 0.07 during pandemic, *p* < 0.001), and RSV (whole year, monthly mean: 6.22 pre-pandemic, 0.29 during pandemic, *p* = 0.007). The seasonality of detection of these viruses in throat swab samples by RT-PCR was lost. The seasonal peak of monthly BoV detection was decreased during the pandemic (season Nov-May, monthly mean: 4.48 pre-pandemic, 1.33 during pandemic, *p* = 0.002). The seasonal peak of CoV detection was delayed by 3 months. The number of RV/EV positive throat swabs did not significantly decrease during the pandemic (whole year, monthly mean: 16.69 pre-pandemic, 14.07 during pandemic, *p* = 0.15), although there were periods with a decreased detection rate of RV/EV in April/May 2020 and January/February 2021. The overall monthly number of PIV detection resulting from molecular diagnostics was reduced during the COVID-19 pandemic (whole year, monthly mean: 3.75 pre-pandemic, 1.21 during pandemic, *p* < 0.001) and it did not peak between April and June 2020. However, there was a peak of PIV detection frequency in December 2020, which corresponded to the seasonal peak before the COVID-19 pandemic.

#### Viral positive respiratory specimens per month after withdrawal of major NPI measures

While the monthly rates of BoV, PIV, and RSV detected in throat swabs by RT-PCR decreased during the COVID-19 pandemic, they increased after withdrawal of major NPI measures (Figure 3). From June through November 2021, the monthly rates of throat swabs tested positive for BoV, CoV, PIV, and RSV significantly exceeded corresponding pre-pandemic monthly detection rates (BoV: 1.3 vs. 10.5 mean monthly positives, *p* < 0.001; CoV: 0.5 vs. 2.7 mean monthly positives, *p* = 0.008; PIV: 3.1 vs. 8.3 mean monthly positives, *p* = 0.007; RSV: 0.4 vs. 19.2 mean monthly positives, *p* < 0.001). Moreover, the increase in the monthly rate of throat swab samples tested positive for BoV, PIV, and RSV between June and November 2021 was temporally unrelated to these viruses' pre-pandemic seasonality. In the case of PIV, the maximum detection rate per month after withdrawal of major NPI measures was comparable to the pre-pandemic maximum (pre-pandemic: 14; July 2021: 14). In the case of RSV, it even exceeded the pre-pandemic maximum (pre-pandemic: 30; October 2021: 51; Figure 1D). Interestingly, several months after withdrawal of major NPI measures, we observed an increase of MPV detection frequency by molecular testing that was in line with the pre-pandemic seasonality of MPV detection (June 2021 to January 2022: *p* = *0.90)*. In comparison to pre-pandemic detection rates, RV/EV detection rate was decreased between July and October 2021 (14.7 vs. 2.5 mean monthly positives; *p* < 0.001), during a period of high detection rates of other respiratory viruses, but showed a peak thereafter which exceeded the pre-pandemic maximum detection rate per month (pre-pandemic: 27; November 2021: 35). We observed no increase of monthly detection of InV in throat swab samples after withdrawal of NPI measures.

### Detection of rotavirus/adenovirus in stool

**Figure 4:**
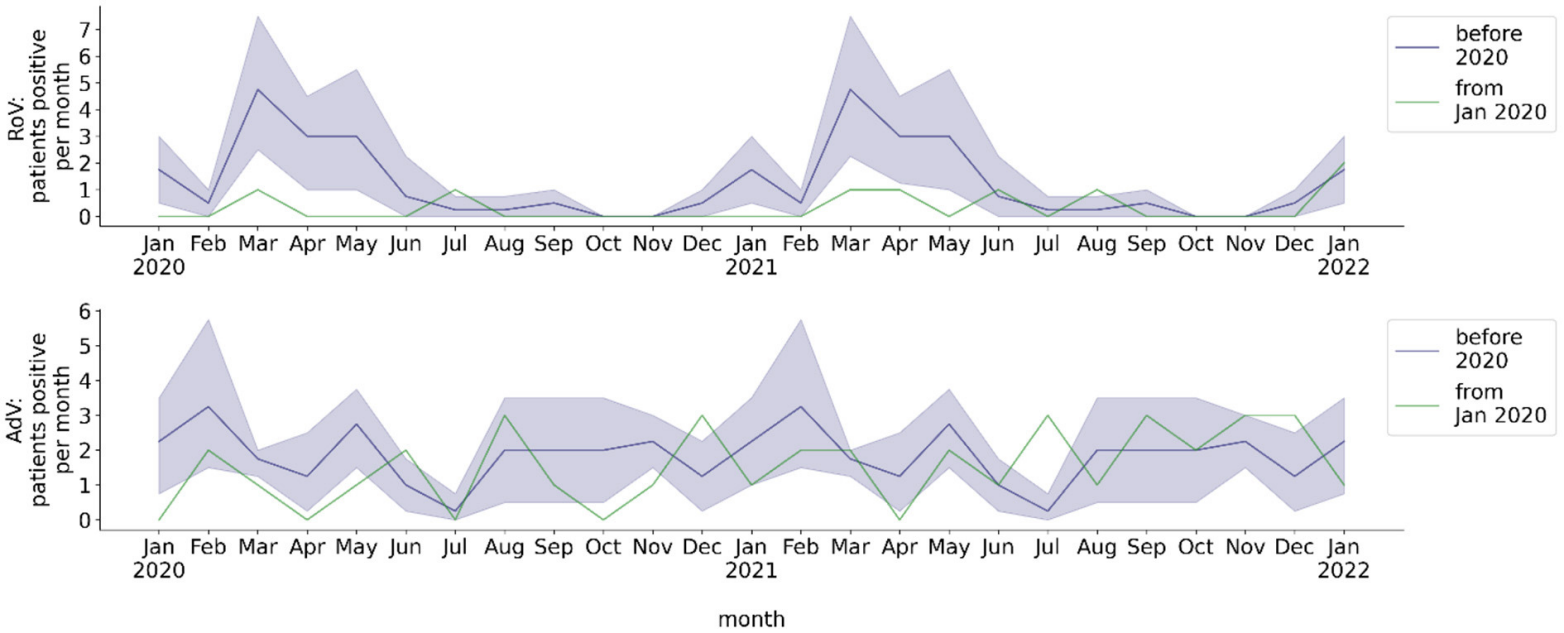
Monthly number of hospitalized patients at BCH positively tested for gastrointestinal viruses. Blue line: mean 2016-2019, blue shade: 95% confidence interval 2016-2019, green line: absolute number per month from 2020, calendar months 2016-2019 are shown as a historical reference and correspond to calendar months, not to year on x-axis. Number of patients per month with stool sample tested positive for RoV or AdV.

The total number of analyzed stool samples was 4,450, total number of patients tested for gastrointestinal RoV/AdV infection was 1,540. The mean total monthly rate of stool EIA/PCR was 28.0 for RoV and 29.2 for AdV before the COVID-19 pandemic, 32.6 for RoV and 28.9 for AdV from the beginning of the pandemic through May 2021, and 47.9 for RoV and 26.6 for AdV from June 2021 to January 2022 (Figures 2B,C). The monthly rate of stool samples tested positive for RoV by EIA/PCR before the pandemic exhibited a seasonal behavior, whereas the rate of stool samples tested positive for AdV by PCR did not (Figure 4). No seasonality of the rate of RoV infection was observed during the COVID-19 pandemic. During the pandemic, the mean monthly rate of patients with stool EIA/PCR tested positive for RoV decreased (season January to May: 0.29, *p* = 0.017), compared to the pre-pandemic period (season January to May: 2.30). In contrast, the monthly number of patients with AdV detected in stool samples by PCR did not decrease during the COVID-19 pandemic (whole year: 1.78 pre-pandemic, 1.29 beginning of pandemic through May 2021, *p* = 0.29). We observed no increase in the monthly detection rate of RoV and AdV by EIA/PCR of stool samples from June 2021 (withdrawal of NPI measures) to December 2021. However, there was a slight increase of the RoV detection rate in January 2022, which temporally corresponded to the pre-pandemic seasonality of RoV detection.

## Discussion

This study describes monthly rates of detection of viral pathogens in pediatric patients admitted to BCH before the COVID-19 pandemic as well as during the COVID-19 pandemic at different levels of public NPI measures.

Detection rates of non-seasonal viruses such as RV/EV in the respiratory tract and AdV in stool showed no change during the period of NPI measures and no immediate change after major NPI withdrawal. In contrast, detection rates of the seasonal viruses BoV, CoV, PIV, and RSV decreased during the period of NPI measures and showed an out of season increase after withdrawal of major NPI measures (Figure 1D). Likewise, rates of detection of the seasonal viruses InV, MPV, and RoV were diminished during the period of NPI measures. However, the detection rates of these viruses, as opposed to those of BoV, CoV, PIV, and RSV, stayed low after withdrawal of major NPI measures or returned to the pre-pandemic seasonality.

Previous studies showed that enveloped viruses such as InV, MPV, PIV, and RSV were detected at a lower frequency in children during the COVID-19 pandemic, whereas non-enveloped viruses such as Rhinoviruses were even detected at a higher frequency (5–7). This was attributed to non-enveloped viruses penetrating, whereas enveloped viruses not penetrating surgical masks (8). In our study we confirmed a stable rate of detection of RV/EV as non-enveloped viruses in children during the COVID-19 pandemic. BoV transmission might be more susceptible to NPI such as FFP2 masks than RV/EV transmission, although Bocaviruses share features with Rhinoviruses, such as the small size and the lack of a viral envelope (Table 1). The seasonal peak of the detection rate of BoV was blunted in winter 2020/21, in a period with an increased strictness of NPI measures in Berlin as a response to high incidence of SARS-CoV-2 infections. Overall, in this study the decline of virus detection frequency in children during the pandemic (April 2020 to May 2021) was related to respiratory viruses with previous seasonality of the detection frequency, regardless of their size or whether they bear a viral envelope.

**Table 1:** Overview of viruses considered in this study.

**Virus**	**Abbreviation in this article**	**Size (nm)**	**Enveloped**
Adenoviruses	AdV	90–100	No
Human Bocavirus	BoV	20	No
Coronaviruses (229E, NL63, HKU1, OC43)	CoV	80–120	Yes
Influenzaviruses	InV	80–120	Yes
Human Metapneumovirus	MPV	150–600	Yes
Parainfluenzaviruses	PIV	150–250	Yes
Respiratory Syncytial viruses	RSV	120–300	Yes
Rhino-/Enteroviruses	RV/EV	20–30	No
Rotavirus	RoV	70	No

Interestingly, we observed an increase of detection rates shortly after withdrawal of major NPI measures only for part of the airborne/droplet transmitted viruses. The detection rate of RSV in children rapidly increased several months before the pre-pandemic RSV season and, in October 2021, even exceeded all pre-pandemic detection rates. This caused a disease burden of pre-school children that possibly surpassed the burden of SARS-CoV-2 infections in this age group during the COVID-19 pandemic. The out of season increase of RSV detection is in line with studies from a number of countries covering five continents (9–14). Several studies also reported rates of infection by RSV that surpassed historical infection rates after public health measures had been reduced (15–17). An increased detection rate of PIV following a period of less frequent detection during the COVID-19 pandemic, as in our study, was described also for the US population (13). This increase occurred in May and June 2021, which corresponded to the pre-pandemic PIV season in the US. In our study we showed an increase of the PIV detection rate specifically for children. In contrast to the US, in our study the peaking PIV detection rate in summer 2021 was clearly delayed by 2 months, in relation the pre-pandemic PIV season. As in the US population, the seasonal peak of CoV detection was also delayed in our study in children by several months (13). To our knowledge, this is the first study that shows a rapid out of season increase of the detection rate of BoV after the withdrawal of major NPI measures.

There are a variety of mechanisms that may account for the out of season increase of detection rates of the described viruses after May 2021. RSV is proposed to interfere with other viruses at the population and host level, which might be a reason for the surge of the RSV infection rate in the absence of InV or Rhinoviruses (18, 19). Another possible explanation, however, is that the rise in the RSV infection rate might be caused by a lack of immunity of children due to missing contact to RSV during pregnancy and early childhood and waning of immunity in older children and the general population, leading to a so-called “immune-debt” (20–24). In other studies, an increasing burden of infection by RSV during the COVID-19 pandemic was not well correlated with school openings (22, 25). In our study, the increase in RSV frequency was temporally associated with several factors: opening of public buildings, schools, restaurants, night life and workplaces, and increasing tourism (Figure 1). On the contrary, the reopening of kindergartens preceded the peak of RSV infection frequency by more than 2 months. This observation is compatible with the thesis that adults and adolescents, not younger children, might be the major reservoir of RSV (21). The concept of an “immune-debt” might also explain the substantial increase in BoV and PIV detection frequency in children in our study after months of NPI measures. The detection rate of RSV during the COVID-19 pandemic was even lower than that of BoV and PIV, and the increase of BoV and PIV detection frequency preceded the one of RSV. Hence, RSV seems more susceptible to the mechanisms described above, including NPI measures. The frequency of InV and MPV detection was even lower than that of RSV during the pandemic and the delay of a surge in detection rates of InV and MPV was even longer than the delay of the current surge of RSV. The detection rate of MPV strongly adhered to a certain seasonality, both before and after the period of NPI measures. This is not well explained by concepts such as the immune debt or viral interference, but rather by changes in social life regarding winter season. Interestingly, in contrast to MPV, InV did not reappear. This might be attributable to the geographical globality of changes in social life and NPI measures. The reappearance of InV will have to be monitored carefully during the following months. In contrast to all other respiratory viruses, the RV/EV detection rate declined in July and surged in November 2021, exceeding pre-pandemic maximum monthly detection rates. Both the decline and the extreme surge thereafter might be a consequence of viral interference, as they were temporally associated with the increase of detection rates of BoV, RSV and PIV and their subsequent decrease.

A decline in the frequency of RoV detection in Germany during the COVID-19 pandemic was previously reported by the Robert Koch Institute, Berlin (26). Our study confirms this observation specifically for children. In contrast to RoV, however, there was no decrease of the rate of AdV detection in the stool of children with diarrhea, compared to the pre-pandemic phase. This might be attributable to AdV not solely relying on fecal-oral transmission and on children as the major reservoir but being transmitted via droplets and surfaces and involving an adult reservoir. In contrast to the respiratory viruses described above, we observed no increase of the RoV detection rate immediately following the withdrawal of major NPI measures. Due to ongoing vaccination against RoV during the COVID-19 pandemic, there might exist less of an “immune-debt,” which might be a reason for the missing surge of the RoV detection rate.

From June 2021 to January 2022 we observed an increased monthly testing rate for respiratory viruses and Rotaviruses (Figures 2A,B). This was temporally associated with an increased detection rate of several respiratory viruses, but also with a subsequent decrease in the detection rate of several viruses. It reflected an increased burden of patients hospitalized due to respiratory and gastrointestinal infections since June 2021. Rather than being an explanation for increased virus detection rates, rising testing rates since June 2021 were a consequence of the increased rate of viral infections. At the beginning of the COVID-19 pandemic, we and other groups observed a decrease in monthly numbers of outpatient presentations (1, 27). Yet this cannot explain the changes in virus detection rates during the pandemic, as patients at BCH only undergo testing when admitted as inpatients to the hospital.

This study also has limitations. It is a single center study including a limited number of patients admitted to the hospital for inpatient care. Multi-center or multinational analyses may prove whether the effects of NPI measures on the seasonality of virus infections we observed here, especially on the detection frequency of BoV and PIV, are transferable to other cities and hospitals. The study includes only the first 8 months after withdrawal of major NPI measures.

In summary, after months of NPI measures to prevent SARS-CoV-2 transmission during the COVID-19 pandemic, withdrawal of major measures led to a surge of infection rates of other viruses such as BoV and RSV in Germany. The infection rates of each of these viruses reached or even surpassed maximum pre-pandemic monthly infection rates. This caused a substantial disease burden in pre-school children. The peaking infection rates of these viruses might be explained by a lack of formation of immunity in infants and waning of immunity in older children and the general population.

## Data availability statement

The raw data supporting the conclusions of this article will be made available by the authors, without undue reservation.

## Ethics statement

Ethical review and approval was not required for the study on human participants in accordance with the local legislation and institutional requirements. Written informed consent from the participants' legal guardian/next of kin was not required to participate in this study in accordance with the national legislation and the institutional requirements.

## Author contributions

NT, NU, VC, MM, and TK conceptualized the study. NT designed instruments for the data analyses, carried out the data analyses, and drafted the initial manuscript. NT, NU, AE, AK, LR, AG, HR, LM, HB, and TK carried out laboratory analyses and collected or provided data. NT, NU, AE, LM, HB, MM, and TK reviewed and revised the manuscript. All authors contributed to the article and approved the submitted version.

## Conflict of interest

Authors NU, AE, AK, and HB were employed by Labor Berlin GmbH, a joint subsidiary of the public hospitals Charité Universitätsmedizin Berlin and Vivantes — Netzwerk für Gesundheit GmbH.

The remaining authors declare that the research was conducted in the absence of any commercial or financial relationships that could be construed as a potential conflict of interest.

## Publisher's note

All claims expressed in this article are solely those of the authors and do not necessarily represent those of their affiliated organizations, or those of the publisher, the editors and the reviewers. Any product that may be evaluated in this article, or claim that may be made by its manufacturer, is not guaranteed or endorsed by the publisher.
